# Role of Saturation and Length of Fatty Acids of Phosphatidylserine in the Aggregation of Transthyretin

**DOI:** 10.1021/acschemneuro.3c00357

**Published:** 2023-09-07

**Authors:** Abid Ali, Kiryl Zhaliazka, Tianyi Dou, Aidan P. Holman, Dmitry Kurouski

**Affiliations:** † Department of Biochemistry and Biophysics, Texas A&M University, College Station, Texas 77843, United States; ‡ Department of Entomology, Texas A&M University, College Station, Texas 77843, United States; § Department of Biomedical Engineering, Texas A&M University, College Station, Texas 77843, United States

**Keywords:** transthyretin, phosphatidylserine, oligomers, fibrils, AFM-IR, LDH

## Abstract

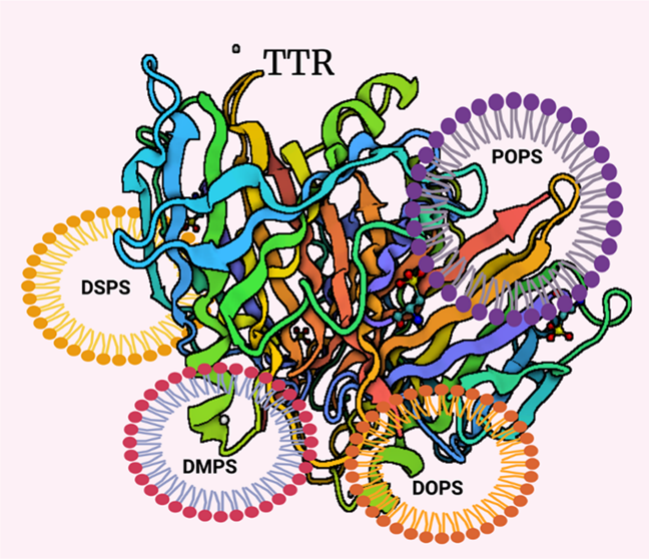

The progressive accumulation of transthyretin (TTR), a small protein that transports thyroxine, in various organs and tissues is observed upon transthyretin amyloidosis, a severe pathology that affects the central, peripheral, and autonomic nervous systems. Once expressed in the liver and choroid plexus, TTR is secreted into the bloodstream and cerebrospinal fluid. In addition to thyroxine, TTR interacts with a large number of molecules, including retinol-binding protein and lipids. In this study, we examined the extent to which phosphatidylserine (PS), a phospholipid that is responsible for the recognition of apoptotic cells by macrophages, could alter the stability of TTR. Using thioflavin T assay, we investigated the rates of TTR aggregation in the presence of PS with different lengths and saturation of fatty acids (FAs). We found that all analyzed lipids decelerated the rate of TTR aggregation. We also used a set of biophysical methods to investigate the extent to which the presence of PS altered the morphology and secondary structure of TTR aggregates. Our results showed that the length and saturation of fatty acids in PS uniquely altered the morphology and secondary structure of TTR fibrils. As a result, TTR fibrils that were formed in the presence of PS with different lengths and saturation of FAs exerted significantly lower cell toxicity compared with the TTR aggregates grown in the lipid-free environment. These findings help to reveal the role of PS in transthyretin amyloidosis and determine the role of the length and saturation of FAs in PS on the morphology and secondary structure of TTR fibrils.

Transthyretin (TTR) is a small 55 kDa protein that transports the thyroid hormone thyroxine in the blood and cerebrospinal fluid.^1,2^ TTR can aggregate forming amyloid oligomers and fibrils. Progressive accumulation of TTR aggregates in the heart leads to the weakening of the heart muscle.^3,4^ This progressive pathology, also known as senile systemic amyloidosis, is typically diagnosed by postmortem exams of patients over 80 years old.^5,6^ Extracellular deposition of TTR aggregates in the peripheral nerves and heart causes familial amyloidotic polyneuropathy and familial amyloidotic cardiomyopathy, respectively.^7,8^ In the former case, irreversible impairments of the nerve functions, including sexual impotence, diarrhea, constipation, and problems with urination, are observed.^4,9^ In the latter case, TTR aggregation causes abnormal heartbeat (arrhythmia) and a sharp drop in blood pressure upon standing.^10,11^ Currently, there is no effective cure for transthyretin amyloidosis. Therefore, it becomes important to determine molecular mechanisms that trigger abrupt protein aggregation in various organs and tissues.^12−16^

A growing body of evidence indicates that lipids can play an important role in protein aggregation.^17−27^ Zhaliazka and co-workers demonstrated that zwitterionic lipids, such as phosphatidylcholine (PC) and phosphatidylethanolamine (PE), inhibited the aggregation of insulin and lysozyme.^23,24,28,29^ In their presence, proteins formed small oligomers that were dominated by the unordered protein. These oligomers exerted significantly lower cell toxicity compared with insulin fibrils formed in the lipid-free environment. Matveyenka and co-workers found that anionic lipids such as cardiolipin (CL), phosphatidylserine (PS), and phosphatidic acid (PA), on the contrary, strongly accelerated the aggregation of both insulin and lysozyme, yielding structurally and morphologically different fibrils compared to those formed in the lipid-free environment.^18,23,24^ Galvagnion and co-workers demonstrated that lipids uniquely alter the aggregation rates of α-Synuclein (α-Syn), a small protein that is localized in the synaptic clefts of neurons.^25−27^ The abrupt aggregation of this protein is directly related to Parkinson’s disease. Galvagnion and co-workers showed that the change in the protein aggregation rate directly depended on the protein-to-lipid ratio. It was proposed that at low concentrations of lipids relative to the concentration of proteins, surfaces of lipid vesicles serve as nucleation sites that enhance the probability of protein interactions.^25−27^ Such interactions are the key step in the protein aggregation. However, with the increase in the concentration of lipids relative to the concentration of proteins, the probabilities of such protein–protein interactions are lowered due to the increasing surface of lipid vesicles. Experimental results reported by Dou and co-workers demonstrated that lipids not only altered the rates of α-Syn aggregation but also uniquely modified the secondary structure of protein oligomers formed in the presence of both PC and PS.^30,31^ Similar results were reported by Zhang and co-workers. The researchers found that low levels of anionic lipids promoted the aggregation of the amyloid precursor peptide (IAPP).^32^ At the same time, the zwitterionic lipid did not alter the rate of IAPP aggregation, whereas cholesterol at or below physiological levels significantly decelerated protein aggregation as well as lowered the propensity of IAPP aggregates to cause membrane leakage. Recently, Zhaliazka and co-workers found that lipids could uniquely alter the structure of amyloid β1–42 (Aβ_1–42_) oligomers and fibrils.^33^ These aggregates exerted significantly higher cell toxicity compared with the Aβ_1–42_ oligomers and fibrils formed in the lipid-free environment.

Experimental results reported by Matveyenka and co-workers demonstrated that not only the net charge of the lipid but also the length and saturation of fatty acids (FAs) play an important role in the rate of protein aggregation.^19,20^ Specifically, it was found that CL with unsaturated FAs enabled much stronger acceleration of insulin aggregation compared with CL with the fully saturated FAs. Furthermore, morphologically and structurally different fibrils were formed in the presence of unsaturated vs saturated CL.^19,20^ It was also reported that PS with 14-carbon-long FAs accelerated insulin aggregation faster than PS with 16- and 18-carbon-long FAs.^20^

Under physiological conditions, PS is localized to the inner membrane.^34^ However, upon cell malfunctioning, PS is transferred to the outer surface of the plasma membrane.^20,35^ We hypothesized that if such malfunctioning cells are not timely removed by macrophages, PS in their membranes can trigger the aggregation of misfolded proteins. In the case of TTR, PS in the outer membrane can trigger TTR amyloidosis. Expanding upon this, we utilize thioflavin T (ThT) assay to investigate the extent to which the length and saturation of FAs in PS alter the rate of TTR aggregation. We also utilize a set of biophysical methods to determine the morphology and secondary structure of TTR fibrils formed in the presence of PS with fully saturated FAs: 1,2-dimyristoyl-*sn*-glycero-3-phospho-l-serine (14:0, DMPS) and 1,2-distearyl-*sn*-glycero-3-phospho-l-serine (18:0, DSPS) as well as PS with unsaturated FAs: 1,2-dioleoyl-*sn*-glycero-3-phospho-l-serine (18:1, DOPS) and 1-palmitoyl-2-oleoyl-*sn*-glycero-3-phospho-l-serine (16:0–18:1, POPS), as shown in Scheme 1. Finally, we use the N27 mice neuronal cell line to examine the toxicity of TTR:DMPS, TTR:DSPS, TTR:POPS, and TTR:DOPS fibrils.

**Scheme 1:**
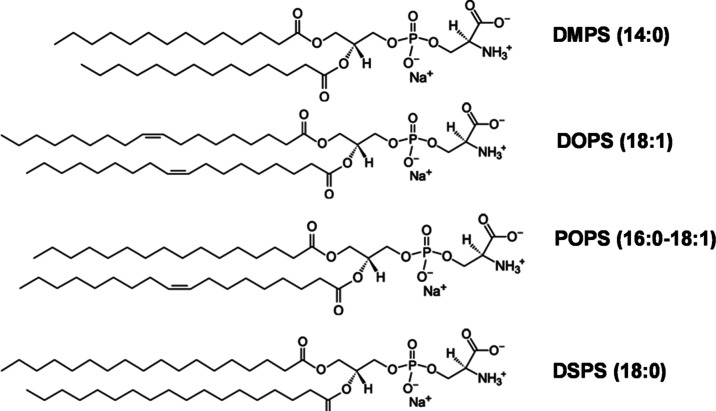
Molecular Structures of DMPS, DOPS, POPS, and DSPS

## Results and Discussion

**Figure 1:**
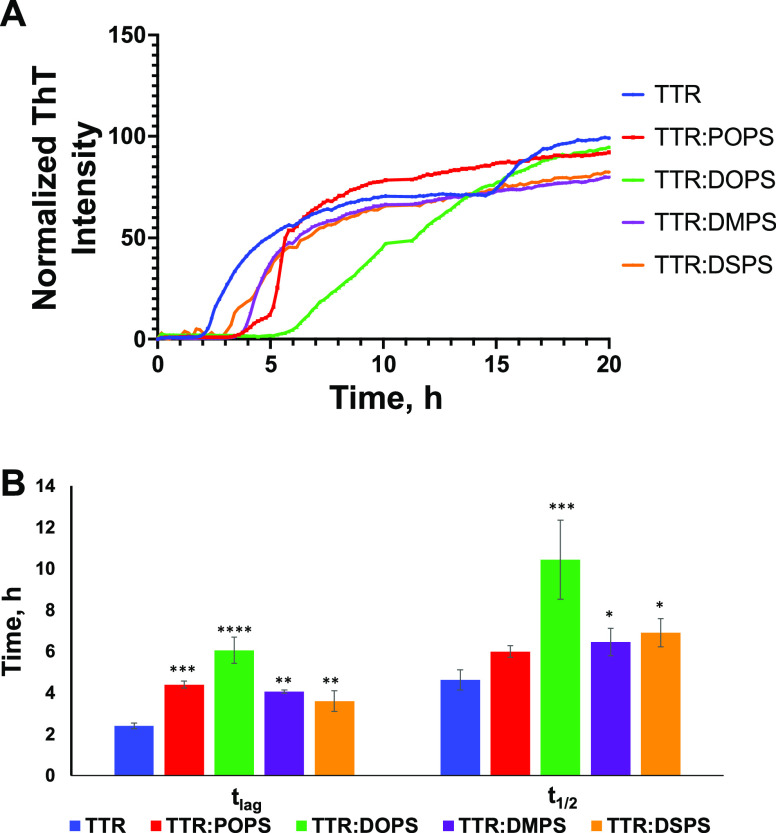
Length and saturation of FAs in PS uniquely alter the rate of TTR aggregation. (A) ThT aggregation kinetics with (B) the corresponding values of *t*_lag_ and *t*_1/2_ of TRR in the lipid-free environment (blue) as well as in the presence of POPS (red), DOPS (green), DMPS (purple), and DSPS (orange). Each kinetic curve is the average of three independent measurements. **P < 0.01, ***P < 0.001, and ****P < 0.0001, according to two-way analysis of variance (ANOVA) and the Tukey honest significant difference (HSD) post hoc test.

First, we used the ThT assay to investigate the extent to which the length and saturation of FAs in PS uniquely alter the rate of TTR aggregation. In the lipid-free environment, TTR aggregates exhibit a well-defined lag-phase (*t*_lag_ = 2.39 ± 0.13 h), which is followed by a rapid increase in the ThT fluorescence, as shown in Figures 1 and S1. The increase in the ThT signal indicates the formation of amyloid fibrils. We found that PS strongly decelerated the rate of TTR aggregation. Specifically, in the presence of DSPS and DMPS, TTR aggregation exhibited *t*_lag_ = 3.4 ± 0.36 h and *t*_lag_ = 4.05 ± 0.08 h, respectively, as shown in Figures 1 and S1. We observed even greater deceleration of TTR aggregation in the presence of DOPS, which resulted in the shift of *t*_lag_ to 6.04 ±0.63 h. We found that TTR exhibited similar *t*_lag_ in the presence of POPS (*t*_lag_ = 4.3 ± 0.16 h) and DMPS (*t*_lag_ = 4.05 ± 0.08 h). Based on these results, we can conclude that unsaturated DOPS with 18-carbon-atom-long FAs enabled greater deceleration of TTR aggregation compared with the fully saturated DSPS (18:0).

We also found that PS with different lengths and saturation of FAs uniquely altered the rate of TTR aggregation. Specifically, DMPS and DSPS exerted similar effects decelerating the rate of TTR aggregation (*t*_1/2_ = 6.44 ± 0.66 and 6.73 ± 0.81 h, respectively). We also found that DOPS exhibited the strongest effect on the rate of TTR aggregation shifting it from *t*_1/2_ = 5.02 ± 1.20 h (TTR) to *t*_1/2_ = 10.14 ± 2.23 h (TTR: DOPS). Finally, we found that POPS did not significantly alter the rate of TTR aggregation. Based on these findings, we can conclude that the length and saturation of FAs in PS uniquely alter the rate of TTR aggregation.

**Figure 2:**
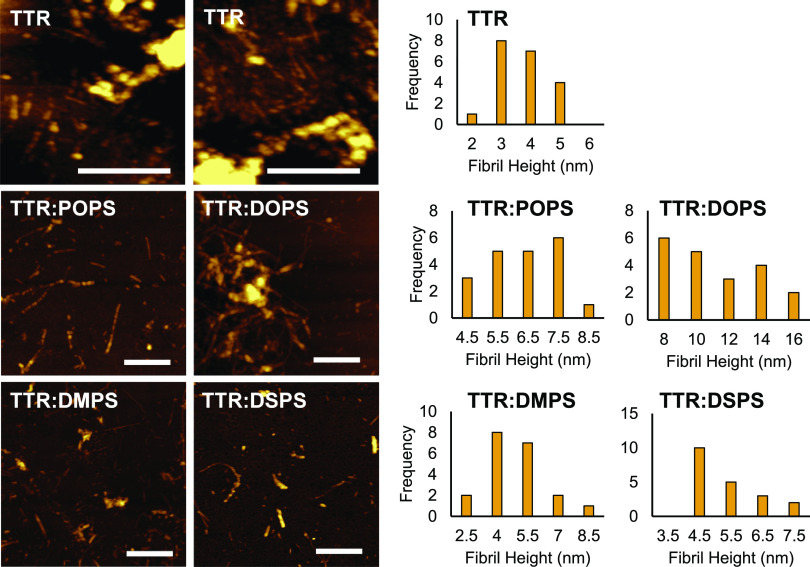
Length and saturation of FAs in PS uniquely alter the morphology of TTR aggregates. AFM images (right) with corresponding height histograms (left) of TTR aggregated in the lipid-free environment and in the presence of POPS, DOPS, DMPS, and DSPS. Scale bars are 500 nm.

Next, we utilized atomic force microscopy (AFM) to examine the topology of TTR aggregates formed in the lipid-free environment and in the presence of PS with different lengths and saturation of FAs, as shown in Figure 2.

We found that in the lipid-free environment, TTR formed thin bead-like fibrils that were 4–5 nm in height, as shown in Figure 2. At the same time, the vast majority of the observed specimens were oligomers with a height distribution from 2 to 5 nm. In the presence of POPS, TTR formed much longer and thicker fibrils. Some of them had smooth topologies, whereas others were composed of small bead-like oligomers. Even thicker fibrils were observed in TTR:DOPS. These aggregates had a smooth topology with heights ranging from 8 to 16 nm. We also found that in the presence of both DMPS and DSPS, TTR formed thin fibrils that had similar heights to TTR fibrils formed in the lipid-free environment. However, we found that similar to TTR:POPS and TTR:DOPS, these aggregates exhibited smooth topologies without a clear visible bead-like structure. Based on these results, we can conclude that the length and saturation of FAs in PS uniquely altered the topology of TTR aggregates formed in the presence of such lipids. Specifically, our findings suggest that lipids facilitate the growth of fibrils, which results in the formation of long fibrillar structures that were largely absent in the lipid-free environment.

**Figure 3:**
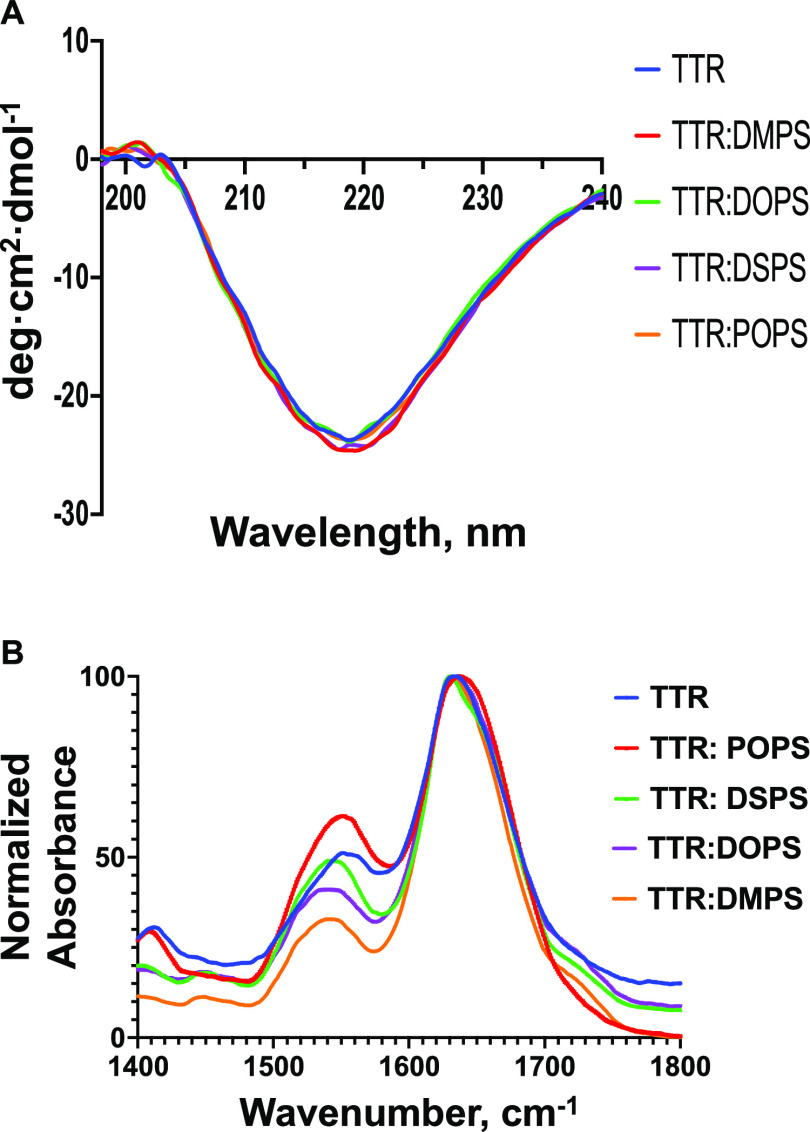
Elucidation of the secondary structure of TTR fibrils formed in the presence of FAs and in the lipid-free environment. CD (A) and FTIR (B) spectra acquired from TTR (blue), POPS (red), DOPS (purple), DMPS (orange), and DSPS (green).

We utilized circular dichroism (CD) and Fourier transform infrared (FTIR) spectroscopy to investigate the secondary structure of TTR fibrils formed in the lipid-free environment and in the presence of PS with the different lengths and saturation of FAs, as shown in Figure 3. In the monomeric state, TTR is a tetramer that is dominated by an α-helical secondary structure.^12−16^ CD spectra acquired from TTR aggregates had a single trough centered around 218 nm. This indicates the predominance of β-sheet-rich aggregates in the analyzed protein samples.^19^ This conclusion could be also supported by the FTIR spectra acquired from TTR fibrils formed in the lipid-free environment and in the presence of POPS, DOPS, DMPS, and DSPS. In the acquired spectra, we observed both amide I (1610–1700 cm^–1^) and amide II (1530–1560 cm^–1^) bands.^19^ The amide I band is centered at ∼1632 cm^–1^, which points to the predominance of β-sheet-rich aggregates in the analyzed samples.^36,37^ It should be noted that both CD and FTIR probe the bulk volume of the analyzed protein samples and, therefore, cannot be used to resolve the secondary structure of individual protein aggregates.^36^

**Figure 4:**
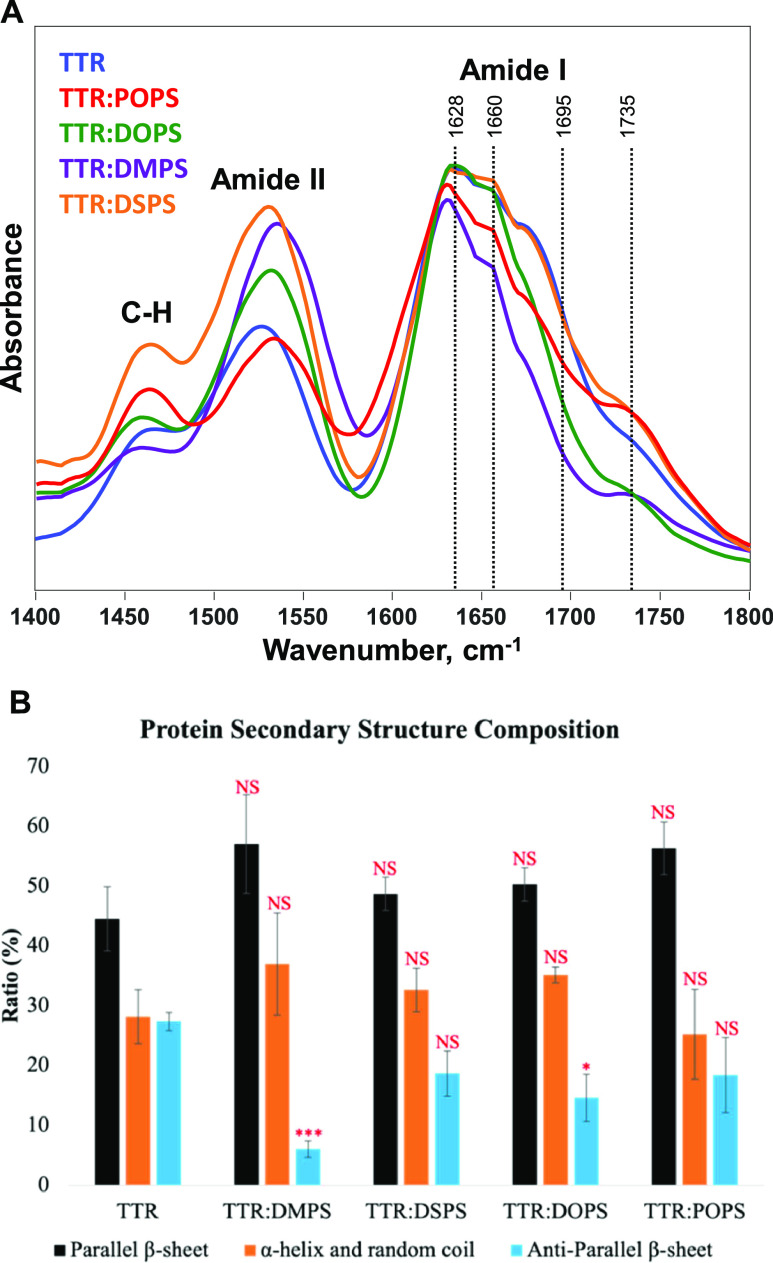
Averaged nano-IR spectra (A) acquired from TTR fibrils formed in the presence of TTR (blue), POPS (red), DOPS (purple), DMPS (orange), and DSPS (green). A histogram (B) that summarizes the distribution of protein secondary structure in the protein aggregates according to the fitting of the amide I band. Parallel β-sheet (1624 cm^–1^) in black, α-helix and random coil (1660 cm^–1^) in orange, and anti-parallel β-sheet (1695 cm^–1^) in light blue. NS, nonsignificant difference, *P < 0.05, **P < 0.01, and ***P < 0.001, according to a nonparametric Kruskal–Wallis test (KKW).

To overcome this limitation, we utilized nano-infrared spectroscopy to analyze the secondary structure of individual TTR fibrils formed in the presence of POPS, DOPS, DMPS, and DSPS as well as in the lipid-free environment, as shown in Figures 4 and S2–S6.^38−40^

Nano-IR confirmed the similarities in the secondary structure of TTR fibrils formed in the presence of PS compared to the TTR aggregates formed in the lipid-free environment. Specifically, TTR fibrils had the same amounts of unordered protein and parallel β-sheet compared to TTR:DMPS, TTR:DSPS, TTR:DOPS, and TTR:POPS.^28^ At the same time, TTR:DMPS and TTR:DOPS fibrils possessed a significantly lower amount of anti-parallel β-sheet compared with TTR fibrils.

Based on these results, we can conclude that the length and saturation of FAs in PS uniquely altered the secondary statute of TTR fibrils formed in the presence of such lipids.

**Figure 5:**
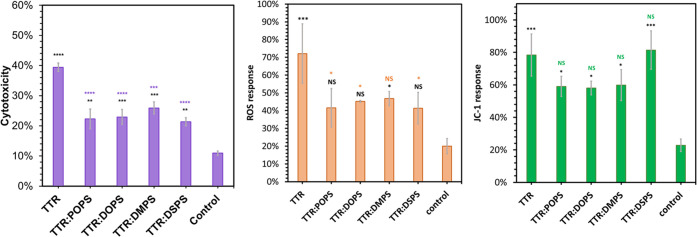
Histograms of lactate dehydrogenase (LDH) (left), reactive oxygen species (ROS) (middle), and JC-1 (right) assays reveal differences between the cell toxicity of TTR, TTR:POPS, TTR:DOPS, TTR:DMPS, and TTR:DSPS. Black asterisks (*) show a significant level of difference between protein aggregates and the control; purple (LHD), orange (ROS), and green (JC-1) asterisks (*) show a significant level of difference between TTR and TTR aggregates formed in the presence of lipids; *P < 0.05, ***P < 0.001, and ****P < 0.0001. NS, nonsignificant difference according to one-way ANOVA and the Tukey HSD post hoc test.

The question to ask is whether the observed structural differences between TTR and fibrils formed in the presence of PS have any biological significance. To answer this question, we investigated the extent to which these protein aggregates exert cell toxicity to rat midbrain dopaminergic N27 cell line, as shown in Figure 5.

The LDH assay revealed a statistically significant difference between the toxicity exerted by TTR fibrils grown in the PS-free environment and protein aggregates grown in the presence of POPS, DOPS, DMPS, and DSPS. Specifically, we found that toxicities of TTR:POPS, TTR:DOPS, TTR:DMPS, and TTR:DSPS fibrils were lower than the toxicity of TTR fibrils. These results demonstrate that the presence of PS in the TTR fibrils reduces the toxicity of TTR aggregates formed in their presence.

Amyloid fibrils exert their toxicities by enhancing ROS levels in cells as well as by damaging cell mitochondria. Expanding upon this, we investigated the extent to which TTR fibrils and TTR:POPS, TTR:DOPS, TTR:DMPS, and TTR:DSPS fibrils alter ROS levels and mitochondrial activity in N27 rat dopaminergic cells, as shown in Figure 5. We found that TTR:POPS, TTR:DOPS, and TTR:DSPS caused significantly lower enhancement of ROS levels compared with TTR fibrils. Our results also showed that all analyzed protein aggregates caused similar levels of mitochondrial dysfunction in the cells.

Our results suggest that the observed differences in the aggregation rate of TTR are linked to the hydrophobic interactions that take place between fatty acids (FAs) of PS and hydrophobic amino acid residues of the protein. Rationalizing upon this, we infer that the length and saturation of FAs in PS alter the binding affinity of phospholipids to TTR.^41^ Previously used NMR and fluorescence methods reveal mechanisms of such lipid–protein interactions for α-Synuclein. It has been found that zwitterionic headgroups of lipids first interact with lysine and glutamic acid residues on the N-terminus (aa 1–60) of a-syn.^42^ Lipid–a-syn interactions are also enhanced by fatty acids of lipids and the central domain (aa 61–95) of α-Syn, also known as the NAC domain.^43,44^ These pieces of experimental evidence suggest that PS can template the aggregation of TTR via both electrostatic and hydrophobic interactions between lipids and proteins. Nevertheless, it remains unclear whether TTR exhibited greater affinity for one of the PS membrane models over the others. Finally, although we hypothesized that the hydrophobic interactions between TTR, POPS, DOPS, DMPS, and DSPS played a major role in the observed differences in the kinetics, secondary structure, and toxicity of TTR, TTR:POPS, TTR:DOPS, TTR:DMPS, and TTR:DSPS aggregates, other properties of PS self-assemblies, such as phase, fluidity, and packing, can be relevant for the results discussed above. Elucidation of all of these factors is the subject for a separate study.

Our findings are in good agreement with the previously reported results by Hou and co-workers.^46,47^ Using the surface plasmon resonance (SPR) biosensor technique, Hou and co-workers found that electrostatic forces primarily influenced the interactions between TTR and anionic lipids. Such electrostatic interactions determined a strong acceleration of the nucleation phase of TTR aggregation. At the same time, our results indicated that anionic PS exhibited the opposite effect on the rate of TTR aggregation. We infer that the observed difference could be attributed to the protonation state of PS. Specifically, the reported experiments by Hou and co-workers were performed at pH 7.4, at which PS possessed a net −1 charge. However, our study was performed at pH 3.0, at which PS was a zwitterion. These comparisons suggest that a net charge of the lipid in addition to its chemical structure is critically important for the observed effect of the lipid on the protein aggregation.

It should be noted that the protein-to-lipid (P:L) ratio is critically important for the changes in the rate of protein aggregation. In our previous study, we demonstrated that lysozyme aggregated much faster at 1:5 and 1:10 P:L ratios compared to 1:1.^45^ Thus, one can expect that with an increase in the concentration of PS relative to the concentration of TTR, greater enhancement of the TTR aggregation rate can be observed.

Hou and co-workers also found that L55P and V30M mutants exhibited a substantially greater binding affinity to the lipids present in the plasma membrane.^46,47^ These differences in protein–lipid interactions could be the underlying molecular cause that explained much greater cell toxicity of L55P and V30M fibrils compared to the WT TTR aggregates. Since both mutants are linked to familial cases of TTR, these results demonstrated that lipid bilayers could play an important role in the triggering of not only WT TTR aggregation but also the aggregation of (significantly more toxic) TTR mutants.

## Conclusions

Summarizing, we found that all analyzed PS strongly decelerated the rate of TTR aggregation. Our results also demonstrate that the length and saturation of FAs in PS uniquely altered the rate of TTR aggregation. The same conclusions could be made for the morphology and secondary structure of TTR aggregates formed in the presence of PS with the different lengths and saturation of FAs. Finally, our results demonstrated that TTR fibrils formed in the presence of PS exerted significantly lower cell toxicity compared with the TTR fibrils formed in the lipid-free environment.

## Methods

### Materials

1,2-Dioleoyl-*sn*-glycero-3-phospho-l-serine (DOPS), 1-palmitoyl-2-oleoyl-*sn*-glycero-3-phospho-l-serine (POPS), 1,2-dimyristoyl-*sn*-glycero-3-phospho-l-serine (DMPS), and 1,2-distearyl-*sn*-glycero-3-phospho-l-serine (DSPS) were purchased from Avanti (Alabaster, AL).

### Liposome Preparation

For the preparation of large unilamellar vesicles (LUVs) of DMPS, POPS, DOPS, and DPSP, 0.6 mg of the lipid was dissolved in 2.6 mL of phosphate buffered saline (PBS), pH 7.4. Next, the solutions were heated in a water bath to ∼50 °C for 30 min. After that, the solutions were immediately immersed into liquid nitrogen for 3–5 min. This heating–thawing cycle was repeated 10 times. Finally, the lipid solutions were passed 15 times through a 100 nm membrane that was placed into the extruder (Avanti, Alabaster, AL). LUV sizes were determined by dynamic light scattering.

### Cloning of Transthyretin (TTR)

The construct corresponding to the TTR amino acid sequence plasmid pcDNA3.1+/C-(K) DYK with Accession No. NM_000371 was purchased from GenScript, USA. The TTR gene was cloned as per the protocol described by Ali et al.^48^ The plasmid pcDNA3.1+/C-(K) was amplified using PCR with this primer set: the primers 5′-ATATATAAGCTTATGGCTTCTCATCGTCTG-3′ tagged with a 5′-*Hin*dIII cleavage site and 5′-ATAT ATCT CGA GT CATTCCT TGGGATTGG-3′ tagged with a 5′ *Xho*I cleavage site. A construct corresponding to the mature protein without the predicted signal sequence was amplified. The PCR-amplified product was double-digested with *Hin*dIII and *Xho*I restriction enzymes. The pET28b vector (GenScript) was also double-digested with *Hin*dIII and *Xho*I restriction enzymes. The digested product (insert and backbone) was ligated using the T4 DNA ligase. The ligated vector was used to transform the *Escherichia coli* DH5α strain, which was plated onto Luria–Bertani (LB) agar plates containing 50 μg/mL of kanamycin (Km). The subsequent transformants were collected for plasmid preparation using Gene Jet Minipreps (Thermo Scientific). The plasmids were digested with *Hin*dIII and *Xho*I (New England Biolabs) to confirm the positive clones. The constructs were sequenced using Eurofins.

### Protein Expression and Purification

TTR was overexpressed in the *E. coli* BL21 (DE3) strain according to the protocol described by Volles and Lansbury.^49,50^ For TTR protein overexpression, LB broth media was used. Briefly, the 1mM IPTG-induced bacterial cells were pelleted down by centrifugation. The pellet was resuspended in lysis buffer (50 mM Tris, pH 8.0, 10 mM EDTA, 150 mM NaCl) with a protease inhibitor cocktail (Roche), sonicated, and followed by heating in a boiling water bath for 20 min. The supernatant was collected after centrifugation (16,000*g*, 30 min). Streptomycin sulfate (10%; 136 μL/mL of supernatant) and glacial acetic acid (228 μL/mL of supernatant) were added to the supernatant, followed by centrifugation (16,000*g*, 4 °C, 10 min). The resulting supernatant was precipitated by an equal volume of saturated ammonium sulfate, prepared at 4 °C. The precipitated protein was washed with a solution of ammonium sulfate (saturated ammonium sulfate and water, 1:1 v/v at 4 °C). The washed pellet was resuspended in 100 mM ammonium acetate and stirred for 10 min. The TTR was precipitated, adding an equal volume of absolute ethanol. Ethanol precipitation was repeated twice at room temperature (RT). The protein was again resuspended in 100 mM ammonium acetate, lyophilized, and stored at −20 °C for further use.

### Size Exclusion Chromatography (SEC)

Purified TTR was dissolved in PBS buffer, pH 7.4. The dissolved protein was treated with thrombin protease to cleave the His-tag with overnight incubation at 4°. The His-tag-cleaved protein was then centrifuged for 30 min at 14,000*g* using a benchtop microcentrifuge (Eppendorf Centrifuge 5424g solution was clear and free of any larger aggregates). Then, 500 μL of concentrated TTR protein solutions was loaded on a Superdex 200 Increase 10/300 gel filtration column attached to an AKTA pure (GE Healthcare) and eluted isocratically at 4 °C in the same buffer with a flow rate of 0.5 mL/min, and 1 ml fractions were collected, as shown in Figure S7.

### Protein Aggregation

In the lipid-free environment, 50 μM TTR was dissolved in sodium acetate 1M KCl buffer. The solution pH was adjusted to pH 3.0 using concentrated HCl. TTR:DMPS, TTR:DSPS, TTR:DOPS, and TTR:DSPS and 50 μM protein were mixed with an equivalent concentration of the corresponding lipid. The pH of the final solution was adjusted to 3.0 using concentrated HCl. Next, the samples were placed into the well plate that was incubated in the plate reader (Tecan, Mannedorf, Switzerland) at 510 rpm for 28h, 37 °C.

### Kinetic Measurements

For the kinetic measurements, ThT was added to the sample to reach the final concentration of 25 μM. Samples were incubated at the same experimental conditions using the same equipment (Tecan, Mannedorf, Switzerland). Fluorescence measurements were taken every 10 min, excitation was 450 nm, and emission was collected at 488 nm.

### Atomic Force Microscopy (AFM) Imaging

We used the AIST-NT-HORIBA (Edison, NJ) AFM system to perform the morphological analysis of protein aggregates. For AFM imaging, silicon tapping-mode AFM probes, AppNano (Mountain View, CA) were used. The force constant was 2.7 N/m; the resonance frequency was 50–80 kHz. For each measurement, an aliquot of the sample was diluted with DI water and placed on the surface of a pre-cleaned glass coverslip. After 20–30 min exposition, the excess solution was removed from the glass surface. Finally, the coverslips were dried under the flow of dried nitrogen. For each sample, 20–30 individual aggregates were measured. Preprocessing of the collected AFM images was performed using AIST-NT software (Edison, NJ).

### Atomic Force Microscopy Infrared Spectroscopy (AFM-IR)

The AFM-IR spectra were collected by the NanoIR3 system (Bruker, Santa Barbara, CA). Gold-coated contact mode scanning probes (NANOANDMORE) were used. More than 15 spectra were collected from more than 10 particles for each sample. Each spectrum was co-averaged by 3 acquisitions by the software. Spectral deconvolution was conducted by GRAM/AI (Thermo Fisher, Houston, TX) to analyze the protein’s secondary structure composition. The statistical analysis for peak fitting was conducted by MATLAB with the Kruskal–Wallis test (KKW).

### Circular Dichroism (CD)

After 48 h of TTR incubation at 37 °C under constant agitation at 510 rpm, samples were diluted to the final concentration of 100 μM using PBS and measured immediately using a Jasco J1000 CD spectrometer (Jasco, Easton, MD). Three spectra were collected for each sample within 190–250 nm and averaged.

### Attenuated Total Reflectance Fourier Transform Infrared (ATR–FTIR) Spectroscopy

After 48 h of incubation at 37 °C, the TTR samples were placed onto the ATR crystal of a 100 FTIR spectrometer (PerkinElmer, Waltham, MA) and dried at room temperature. Three spectra were collected from each sample.

### Cell Toxicity Assays

Rat midbrain dopaminergic N27 cells were grown in RPMI 1640 Medium (Thermo Fisher Scientific, Waltham, MA) with 10% fetal bovine serum (FBS) (Invitrogen, Waltham, MA) in a 96-well plate (10,000 cells per well) at 37 °C under 5% CO_2_. After 24 h, the cells were found to fully adhere. Next, 100 μL of the cell culture was replaced with 100 μL of RPMI 1640 Medium with 5% FBS containing 10 μL of TTR aggregates. After 24 h of incubation with the sample of the protein aggregates, a lactate dehydrogenase (LDH) assay (G1781, Promega, Madison, WI) was used to determine the toxicity of protein aggregates. Absorption measurements were made in a plate reader (Tecan, Mannedorf, Switzerland) at 490 nm.

In parallel, for a reactive oxygen species (ROS) and JC-1 assay, rat midbrain dopaminergic N27 cells were cultured in RPMI 1640 Medium (Thermo Fisher Scientific, Waltham, MA) supplemented with 10% fetal bovine serum (FBS) (Invitrogen, Waltham, MA) in a 96-well plate at a density of 30,000 cells per well. The cells were incubated at 37 °C with 5% CO_2_ until they fully adhered after 24 h. Next, 300 μL of the cell culture medium was replaced with 300 μL of RPMI 1640 Medium containing 5% FBS and 30 μL of TTR aggregates. After incubating with the protein aggregates for 24 h, a ROS and JC-1 assay was performed. The ROS reagent (C10422, Invitrogen) was added to achieve a final concentration of 5 μM. The cells were then incubated at 37 °C with 5% CO_2_ for 30 min. After removing the supernatant, the cells were washed with RPMI 1640 Medium containing 5% FBS. Subsequently, the cells were treated with trypsin and suspended in 200 μL of 1xPBS, pH 7.4. The red channel (λ = 633 nm) of an Accuri C6 Flow Cytometer (BD, San Jose, CA) was used to measure the samples and determine the percentage of ROS-positive cells using Acura software. For JC-1 staining, the cells were treated with JC-1 reagent (M34152A, Invitrogen) to achieve a final concentration of 50 μM and incubated at 37 °C with 5% CO_2_ for 30 min. After removing the supernatant and treating the cells with trypsin, they were resuspended in 200 μL of 1xPBS, pH 7.4. The green channel (λ = 488 nm) of an Accuri C6 Flow Cytometer (BD, San Jose, CA) was used to measure the samples and determine the percentage of cells exhibiting JC-1 staining.

## Supporting Information Available

ThT kinetics of TTR aggregation (Figure S1); AFM-IR spectra acquired from TTR aggregates (Figures S2–S6); and chromatogram of SEC protein purification (Figure S7) (PDF)
