# Supplementary material for: Role of Saturation and Length of Fatty Acids of Phosphatidylserine in the Aggregation of Transthyretin
Source: ACS Chem Neurosci. 2023 Sep 7;14(18):3499–506. doi: 10.1021/acschemneuro.3c00357 (PMC10862486; doi:10.1021/acschemneuro.3c00357)
Supplement: Supplementary file 1 — cn3c00357_si_001.pdf [file cn3c00357_si_001.pdf]

The Role of Saturation and Length of Fatty Acids of Phosphatidylserine in the Aggregation of Transthyretin

Abid Ali,[1] Kiryl Zhaliazka,[1] Tianyi Dou,[1] Aidan P. Holman[2] and Dmitry Kurouski[*1,3]

1. Department of Biochemistry and Biophysics, Texas A&M University, College Station, Texas 77843, United States
2. Department of Entomology, Texas A&M University, College Station, Texas 77843, United States
3. Department of Biomedical Engineering, Texas A&M University, College Station, Texas, 77843, United States

Email: dkurouski@tamu.edu

Supporting Information:

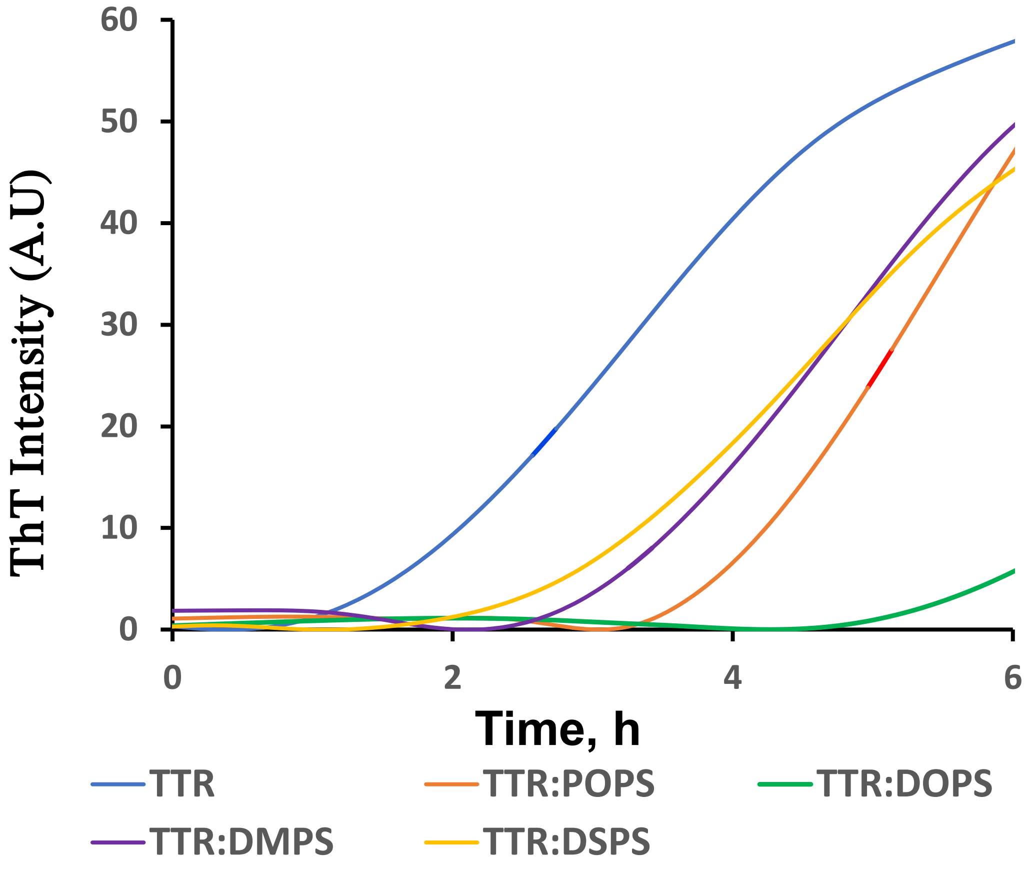

Figure S1. The length and saturation of FAs in PS uniquely alter the rate of TTR aggregation.

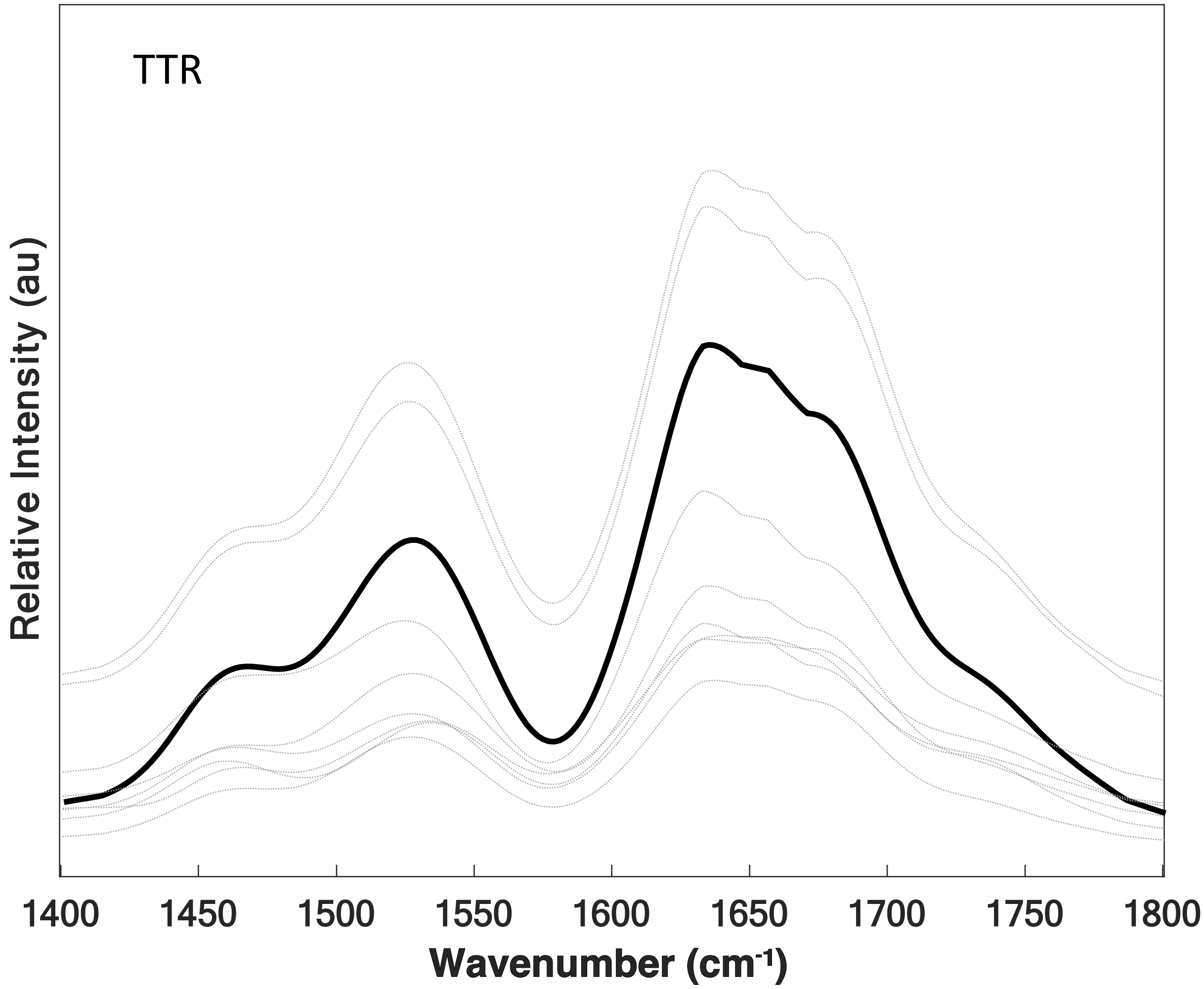

Figure S2. Individual (grey) and averaged (black) AFM-IR spectra acquired from TTR fibrils.

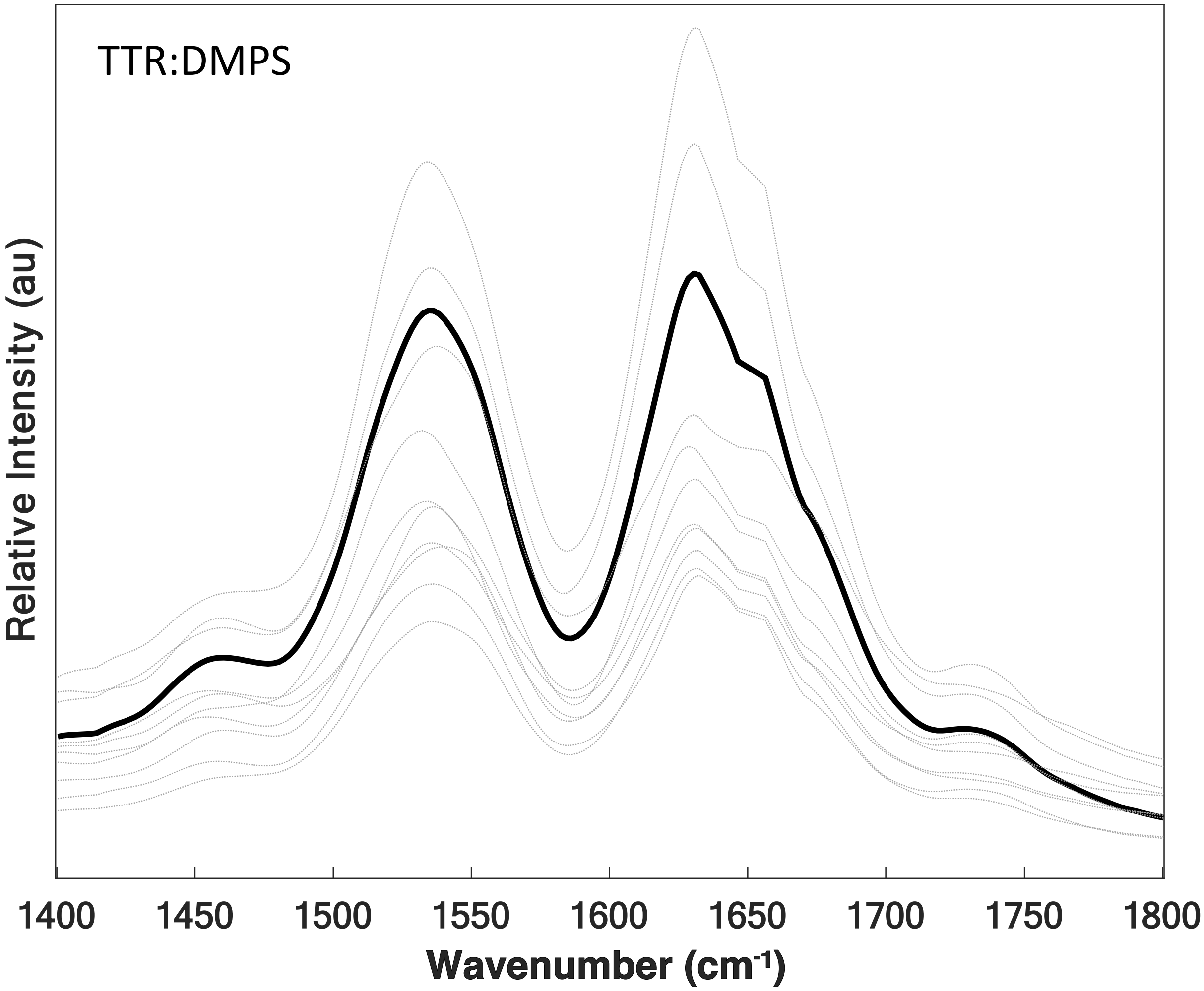

Figure S3. Individual (grey) and averaged (black) AFM-IR spectra acquired from TTR:DMPS fibrils.

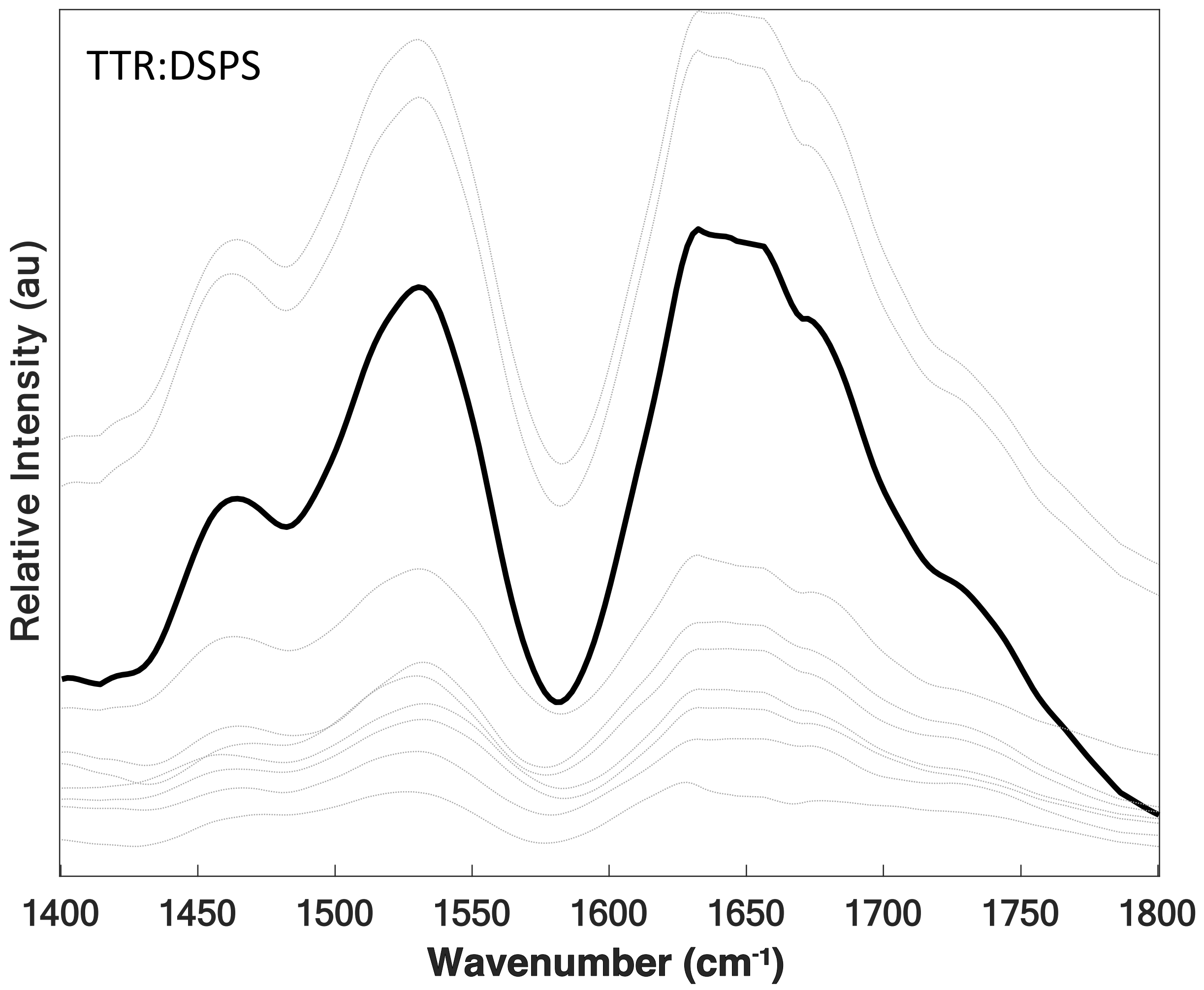

Figure S4. Individual (grey) and averaged (black) AFM-IR spectra acquired from TTR:DSPS fibrils.

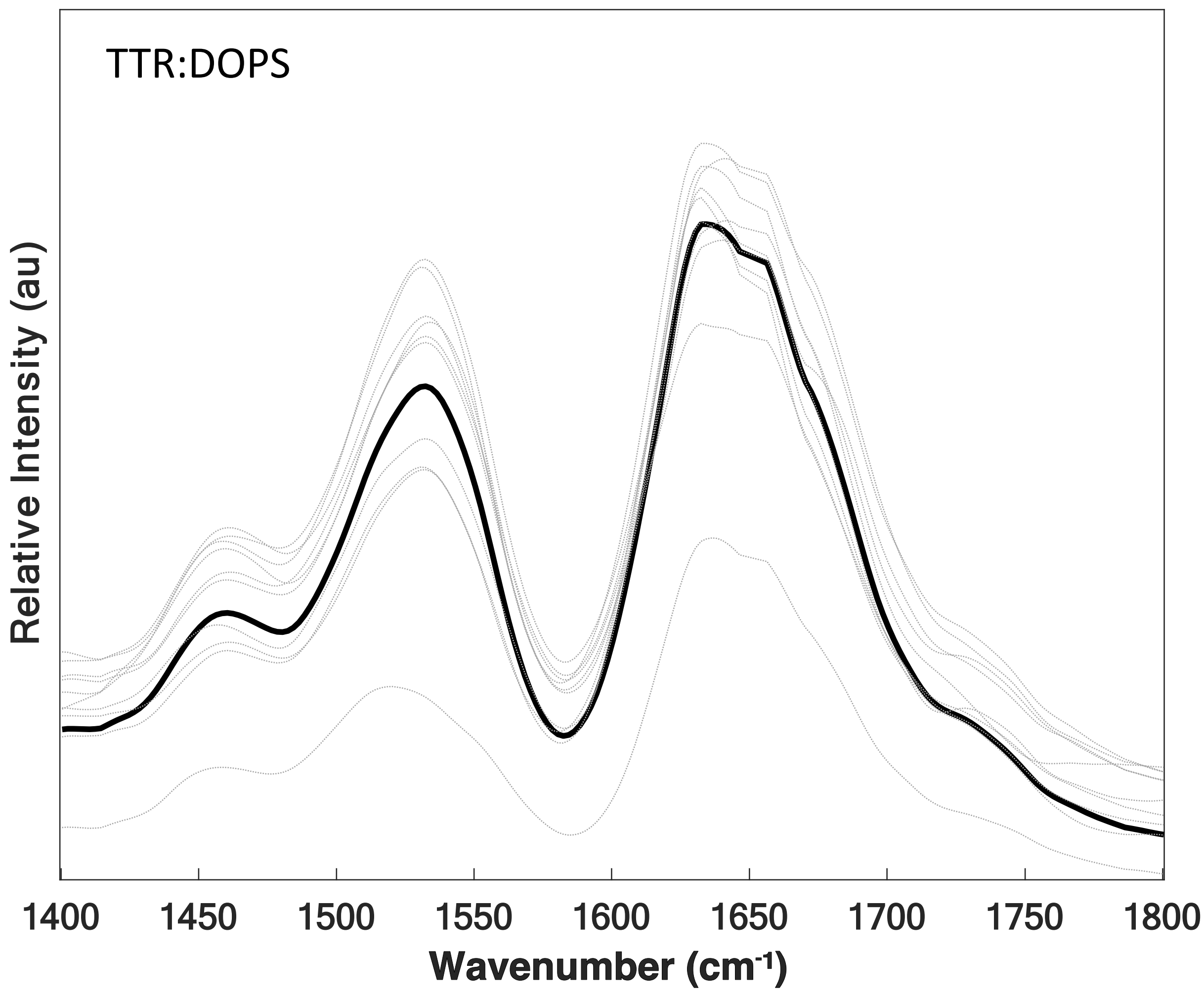

Figure S5. Individual (grey) and averaged (black) AFM-IR spectra acquired from TTR:DOPS fibrils.

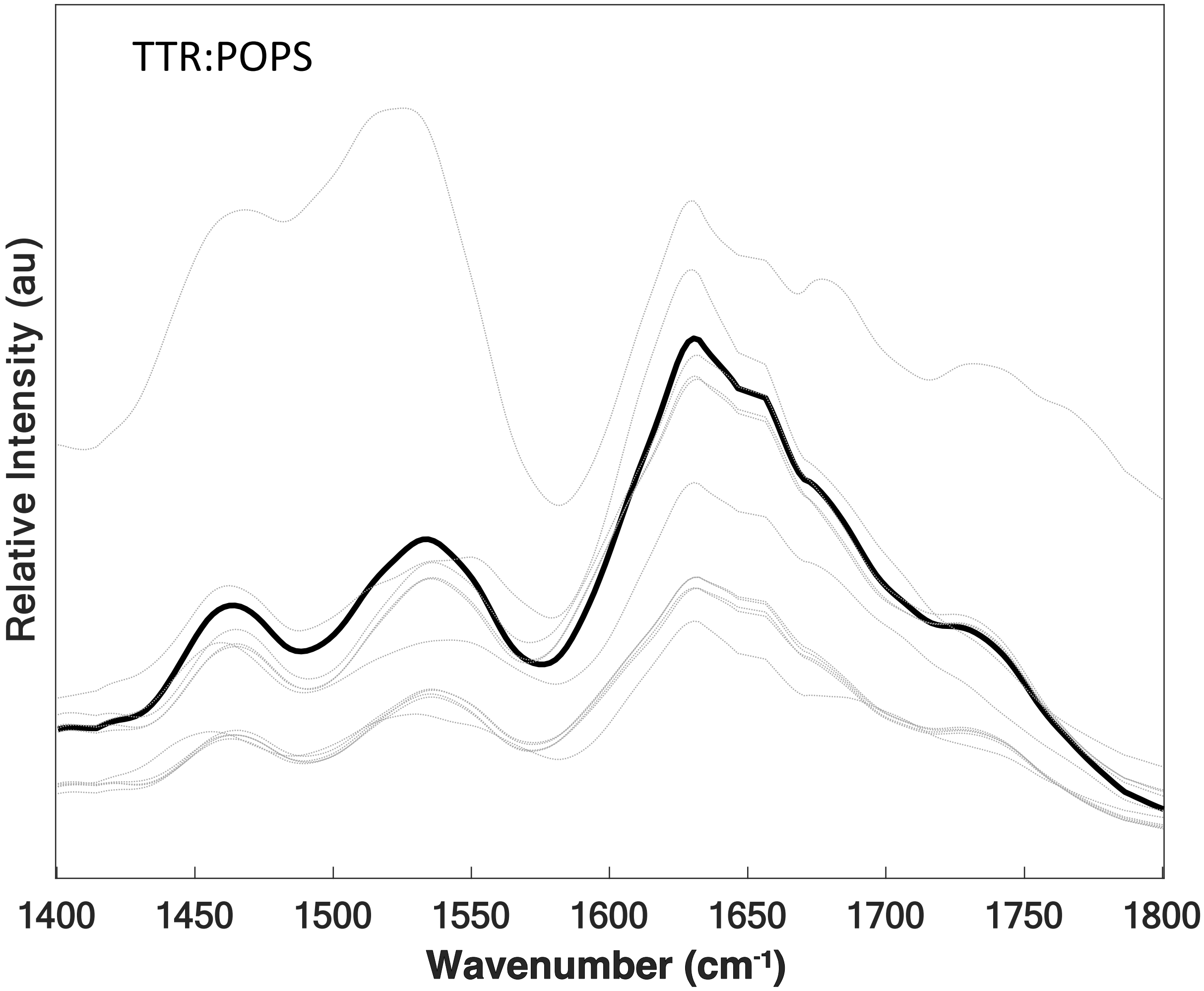

Figure S6. Individual (grey) and averaged (black) AFM-IR spectra acquired from TTR:POPS fibrils.

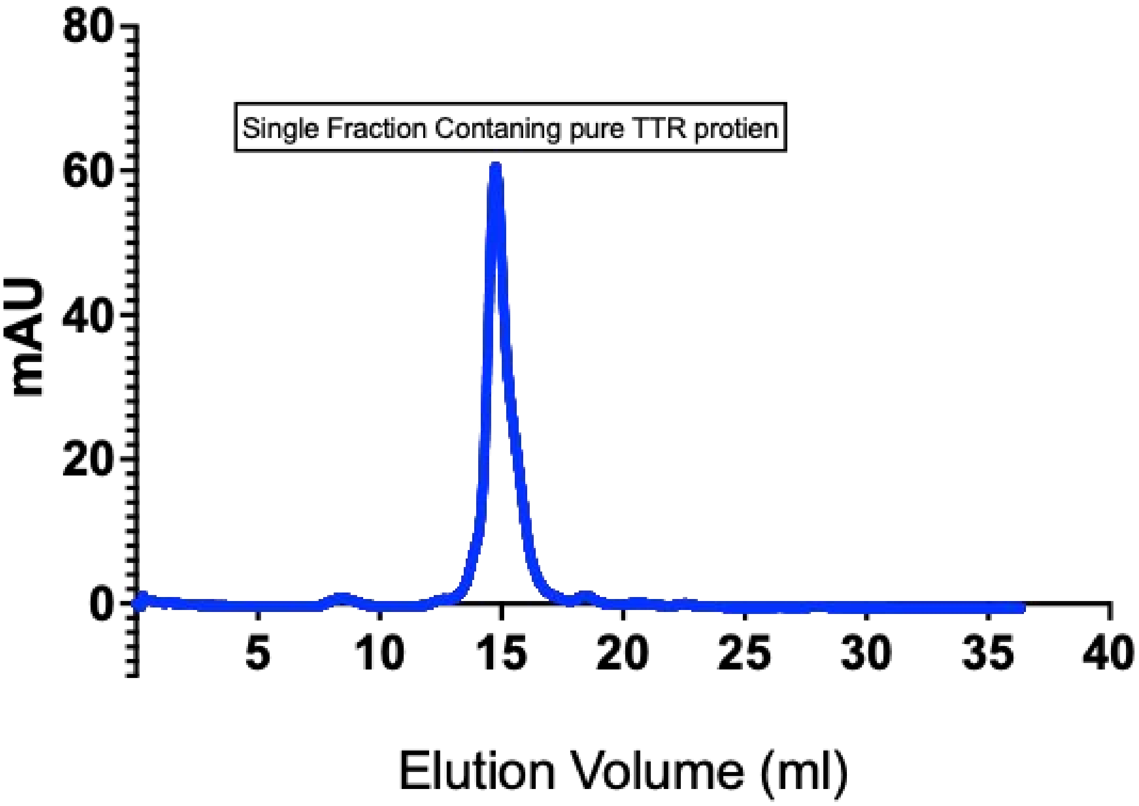

Figure S7. SEC chromatogram of purified TTR.
